# Fermentation and Hydrogen Metabolism Affect Uranium Reduction by Clostridia

**DOI:** 10.5402/2013/657160

**Published:** 2013-02-21

**Authors:** Weimin Gao, Arokiasamy J. Francis

**Affiliations:** ^1^Center for Biosignatures Discovery Automation, Biodesign Institute, Arizona State University, Tempe, AZ 85287, USA; ^2^Division of Advanced Nuclear Engineering, Pohang University of Science and Technology, Pohang 790-784, Republic of Korea; ^3^Environmental Sciences Department, Brookhaven National Laboratory, Upton, NY 11973, USA

## Abstract

Previously, it has been shown that not only is uranium reduction under fermentation condition common among clostridia species, but also the strains differed in the extent of their capability and the pH of the culture significantly affected uranium(VI) reduction. In this study, using HPLC and GC techniques, metabolic properties of those clostridial strains active in uranium reduction under fermentation conditions have been characterized and their effects on capability variance of uranium reduction discussed. Then, the relationship between hydrogen metabolism and uranium reduction has been further explored and the important role played by hydrogenase in uranium(VI) and iron(III) reduction by clostridia demonstrated. When hydrogen was provided as the headspace gas, uranium(VI) reduction occurred in the presence of whole cells of clostridia. This is in contrast to that of nitrogen as the headspace gas. Without clostridia cells, hydrogen alone could not result in uranium(VI) reduction. In alignment with this observation, it was also found that either copper(II) addition or iron depletion in the medium could compromise uranium reduction by clostridia. In the end, a comprehensive model was proposed to explain uranium reduction by clostridia and its relationship to the overall metabolism especially hydrogen (H_2_) production.

## 1. Introduction 

Subsurface contamination by radionuclides and toxic metals is a major problem throughout the U.S. Department of Energy's (DOE's) complex; uranium contamination evokes particular environmental concern due to the high solubility and mobility of its oxidized form, U(VI). As physically removing the contaminated material is financially prohibitive, we need innovative, cost-effective *in situ* stabilization technologies that exploit the processes of natural attenuation. Recently, researchers at some DOE sites have assessed the microbial stabilization of actinides (U, Pu, and Np) and fission products (Tc) in subsurface environments, as in the uranium mill tailing remedial action (UMTRA) site in Rifle, CO (http://www.gjem.energy.gov/moab/) and at the Oak Ridge Field Research Centre (ORFRC) at Oak Ridge, TN (http://www.esd.ornl.gov/orifrc/). A wide variety of bacteria, including *Desulfovibrio*, *Geobacter*, and *Shewanella,* can couple the oxidation of organic compounds to the reduction of U(VI) and thus reductively precipitate uranium in its reduced form U(IV) [1–3]. Investigators have explored the mechanisms of uranium(VI) reduction by anaerobic bacteria [4–6].

Clostridia are anaerobic fermenting bacteria. They are gram-positive, spore-forming bacilli found in soils, sediments, wastes, and in the normal intestinal flora of humans and animals. They play a major role in decomposing a wide variety of organic compounds [7]. The fermentation industry uses them widely to produce solvents and biofuels, including acetone, ethanol, butanol, and hydrogen [8, 9]. Some members of the class also are capable of non symbiotic nitrogen (N_2_) fixation [10], and others can reductively synthesize acetate from carbon dioxide (CO_2_) via the Wood-Ljungdahl pathway [11]. Unlike those anaerobic respiratory bacteria, clostridia classically were viewed as obligate anaerobic, fermentative bacteria, although recent evidence showed that some strains of this group are tolerant of oxygen to some extent [12, 13].

Previously, it was showed that *Clostridium* sp. BC1 (ATCC 53464) and *C. sphenoides* (ATCC 19403) can reduce uranyl-nitrate, -acetate, and -citrate complexes, as well as complexes of other metals [14–16]. We then argued that if the reduction of uranyl compounds could be a general property of clostridia. We have further found that not only is this ability common among more clostridia species, but that also the strains differed in the extent of their capability. The pH of the culture significantly affected uranium(VI) reduction, with pH 5-6 being the optimal one in most cases. Among the strains tested, *Clostridium *sp. BC1 showed the highest rate of U(VI) reduction [17]. Apparently, the evidence suggested that clostridia is one of the major players in uranium reduction *in situ* in an acidic (pH = 4) uranium-mine pit water [18, 19], at a military facility near Chesapeake Bay, Maryland, United States [20], as well as at the ORFRC at Oak Ridge, TN [21]. 

In this study, using HPLC and GC techniques, we have continually characterized metabolic properties of those clostridial strains active in uranium reduction under fermentation conditions and discussed their effects on performance of uranium reduction. We also demonstrated that hydrogen metabolism could play an important role in uranium(VI) and iron(III) reduction by clostridia. In the end, we proposed a comprehensive model to explain molecular mechanisms of uranium reduction by clostridia and its relationship to the overall metabolism especially hydrogen (H_2_) production. 

## 2. Materials and Methods

### 2.1. Bacterial Strains and Media

*Clostridium *sp. BC1 (ATCC 53464) was isolated in our laboratory [22]. We purchased *C. sphenoides* (ATCC 19403), *C. acetobutylicum* (ATCC 824), and *C. pasteurianum* (ATCC 7040) from the American Type Culture Center (ATCC). All strains can ferment glucose, but only *C. sphenoides* can metabolize citric acid as the sole carbon and energy source. All strains were grown anaerobically and maintained in the mineral salts medium, herein called MSM medium, containing per liter: glucose 5.0 g, NH_4_Cl 0.5 g, glycerol phosphate 0.3 g, MgSO_4_·7H_2_O 0.2 g, CaCl_2_·2H_2_O 0.5 g, peptone 0.1 g, and yeast extracts 0.1 g, 6.25 mL of 1.6 mM FeSO_4_·7H_2_O, at pH 6.8. The medium was prepared as follows. We dissolved all the ingredients except FeSO_4_ to about 1000 mL in deionized water in a 2000 Erlenmeyer flask and prereduced by boiling and purging with nitrogen gas for 20 minutes. After allowing the solution to cool, we placed the flask in an anaerobic glove box (95% N_2_, 5% H_2_) and then added to the medium 6.25 mL of 1.6 mM prereduced FeSO_4_·7H_2_O (prepared by dissolving 90 mg ferrous sulfate in 200 mL of prereduced deionized water and adding 0.5 mL concentrated HCl) and made up the total volume to 1000 mL with prereduced deionized water. The pH was adjusted to 6.8 before dispensing 40 mL aliquots of the medium into 60 mL serum bottles, fitted with butyl rubber stoppers, then crimp-sealing them with aluminum caps before autoclaving. *C. sphenoids* (ATCC19403) was also cultured in Simmons Citrate medium (SCM); all materials and their preparation were exactly the same as for the MSM medium, except that we substituted glucose with 8.2 g sodium citrate. To stabilize the pH of the culture, as needed, we added 50 mM PIPES (pH 6.8) or 50 mM MES (pH 6.2) to the medium. The bacterial cultures were grown at 26°C in the dark on a rotary shaker agitated at 100 rpm. Total gas production in the headspace of the sample was measured by a pressure transducer with a needle (Model 665-D/030, Wallace and Tiernan) [22]. We removed four-milliliter aliquots of the culture to determine bacterial growth, change in pH, and production of organic acid metabolites. The growth of the bacterium was measured by recording the turbidity of the medium at 600 nm, using a Bausch and Lomb Spectronic 20 spectrophotometer.

### 2.2. Uranium(VI) Solution Preparation

The U(VI)-nitrate (Uranyl nitrate) stock solution (0.5 M) was made by dissolving solid UO_2_(NO_3_)_2_ into prereduced deionized water. The exact concentration of uranium(VI) stock was calibrated using a KPA machine (Kinetic Phosphorescence Analyzer, Chemchek Instruments, Inc.) that was manipulated according to the manufacturer's instruction. For the 10 mM U(VI)-nitrate stock solution, 5 mL of prereduced deionized water was added to a beaker along with 0.25 mL of 0.5 M U(VI)-nitrate, the pH adjusted to 6.1 with NaOH, and the solution diluted to 10 mL for a final concentration of 10 mM. The complexes were stored in the dark and then readjusted to pH 6.1 after 24 hours, and filter-sterilized (0.45 *μ*m) into a vacutainer tube. 10 mM U(VI)-citrate stock solution was made by mixing the 0.5 M U(VI)-nitrate solution with citrate acid at the molar ratio of 1 : 1 as described below. Briefly, 0.5 mL of prereduced citric acid solution (200 mM) was added to a beaker in the gloved box along with 0.2 mL of 0.5 M U(VI)-nitrate. The pH was adjusted to 6.1 with prereduced NaOH and diluted to 10 mL for a final concentration of 10 mM. 

### 2.3. Uranium(VI) Reduction Assay

Ten milliliters of the culture at the late-log phase was transferred to anaerobic preautoclaved serum bottle (20 mL), and then 0.1 mL of 10 mM U(VI)-nitrate solution added via a 1-mL syringe with a needle. To determine the U(VI) concentration over time, aliquots of 0.1 to 0.2-mL of the bacterial culture were taken from the serum bottle, 5 *μ*L of the culture diluted in 2 mL deionized water and immediately analyzed for U(VI) by KPA. 

To assess the effect of pH on U(VI) reduction by Clostridia, ten milliliter aliquots of the late-log growth phase of the bacterial culture were adjusted to the required pH values using 1 N NaOH or HCl.

### 2.4. UV-Vis Analysis of Uranium(VI) and Uranium(IV)

The UV-vis (ultraviolet-visible) spectrophotometry was used for obtaining absorption spectra of both uranium(VI) and uranium(IV). A Hewlett Packard Model 8453 UV-VIS spectrometer was used for this purpose. The sample was prepared as follows: after completing the uranium reduction assay described above, the total leftover culture in serum bottle was collected into a centrifuge tube in the glove box. Then, its pH was adjusted to 11 using 1 N NaOH, and the culture centrifuged at 10000 rpm for 10 minutes, the supernatant discarded, and the pellet was resuspended in 2-mL 10 mM citric acid solution to extract the uranium species. The solution was filtered through a 0.45 *μ*m membrane and then the filtrate was analyzed by UV-VIS spectrophotometry to determine the absorption spectra of both uranium(VI) and uranium(IV).

### 2.5. Metabolite Profiling

The fermentation products, including organic acids and gases, were analyzed by high performance liquid chromatography (HPLC) and gas chromatography (GC). The HPLC unit consisted of a Shimadzu SCL-10A System Controller, a SIL-10A autoinjector, and a LC-10AS liquid chromatograph. The culture sample, filtered through a 0.45 *μ*m filter, was analyzed by HPLC for organic acids using an SPD-10A UV-vis detector at 210 nm; for glucose, we employed a RID-6A refractive index detector (Shimadzu). A SRI 8610C gas chromatograph fitted with a thermal conductivity detector was used to analyze the H_2_ produced through fermentation. 

## 3. Results

### 3.1. Growth of Bacteria, Gas Production in the Headspace, Changes in pH, and Metabolite Profile

**Figure 1:**
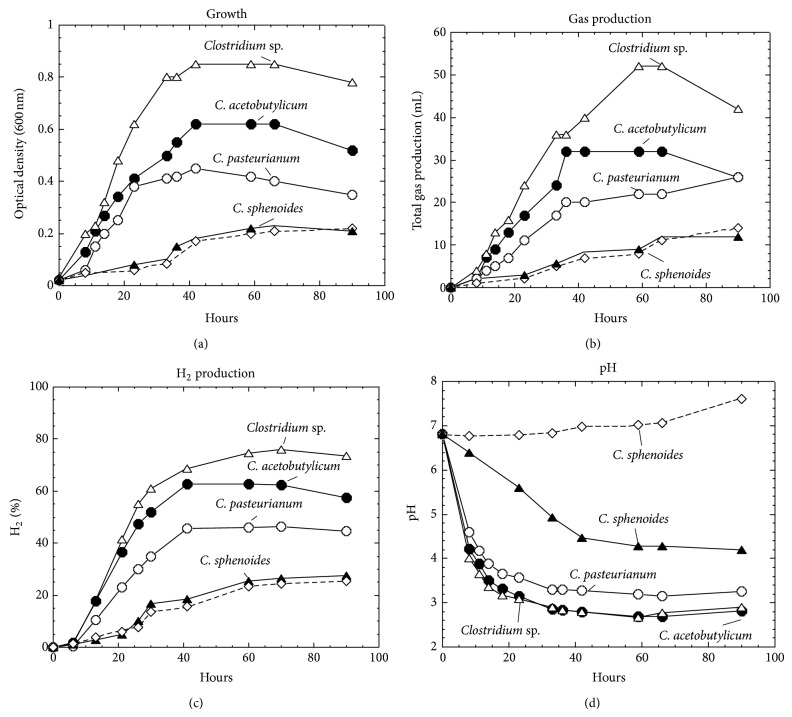
Growth, total gas production, hydrogen production, and pH change in *Clostridia*. Note that all four strains including *Clostridium* sp., *C. acetobutylicum*, *C. pasteurianum,* and *C. sphenoides* were grown in MSM medium, while *C. sphenoides* was also in SCM medium (marked with dashed line).

We determined the rate of growth, changes in pH of the media, and gas production of the four clostridia strains cultured in MSM or SCM. We also analyzed the extent of consumption of the carbon source and the metabolic products from the strains.  Figure 1 shows the highest cell density (i.e., rate of growth) in *Clostridium* sp.; this strain also generated the most gas, even after entering stationary phase, while all other strains stopped producing gas at the stationary phase. Indeed, the growth of all other isolates in the MSM medium was poorer. The pH fell from 6.8 to below 3.0 in *Clostridium* sp., *C. acetobutylicum*, and *C. pasteurianum,* and to 4.2 in *C. sphenoides*. This drop probably reflected the production of organic acids (the drop might be caused also by changing the activity of H-ATPase). GC analysis of the headspace gas showed that the maximum percentage of hydrogen (H_2_) produced by *Clostridium* sp., *C. acetobutylicum*, *C. pasteurianum,* and *C. sphenoides,* respectively, was about 75%, 60%, 45%, and 25% (Figure 1). Growth of *C. sphenoides* in SCM medium containing citrate as the carbon source and comparing it with that in MSM medium revealed no significant differences in its maximum cell density and gas production (Figure 1). However, the medium's pH slightly increased from near neutral to around pH 7.8, due to the concurrent consumption of citric acid (Figure 1).

**Figure 2:**
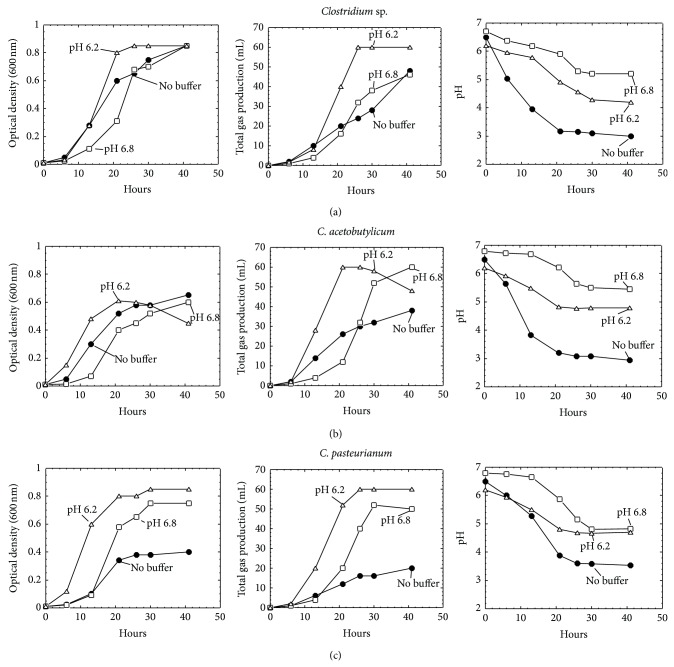
Growth, headspace gas production, and pH change in clostridia strains cultured in MSM medium supplemented with and without pH buffer. The initial pH of the medium is indicated.

We tested alternative carbon sources including sucrose and glycerol and found that neither improved the growth of these strains (data not shown). However, growing the clostridia in media supplemented with a pH buffer to stabilize pH alleviated the drop in pH due to the production of organic acid during glucose fermentation (Figure 2). When cultured in MSM medium supplemented with 50 mM PIPES (pH 6.8), pH of the medium reached around 5 to 6 by the late-log phase of growth for *Clostridium* sp., *C. acetobutylicum,* and *C. pasteurianum*; supplementation with 50 mM MES (pH 6.2) caused the final pH of these cultures to reach 4 to 5. Furthermore, the pH-buffered medium affected the growth kinetics and final cell density of the strains. Compared to growth in MSM medium without pH buffer, *Clostridium* sp. grew much slower initially when the medium was buffered to pH 6.8, although growth later accelerated and it reached the stationary phase at the same time (40 hours) and at the same optimal density (OD_600 nm_ = 0.8). In contrast, *Clostridium* sp. grew much faster in the medium buffered to pH 6.2, reaching the stationary phase 20 hours earlier at the same optimal density (OD_600 nm_ = 0.8) (Figure 2).

The growth of *C. acetobutylicum* was higher in medium buffered at pH 6.2, and it attained the stationary stage in about 10 hours earlier than that of unbuffered medium. By contrast, in MSM buffered to pH 6.8, like *Clostridium* sp., its growth was much lower at the first 15 hours; thereafter, it reached the same level as that in unbuffered culture. Addition of buffers did not result in an increase in final cell density (Figure 2). The growth of *C. pasteurianum* increased in the pH 6.8 and 6.2 adjusted buffered medium (Figure 2). The similar trends also occurred in the production of total gas by these strains (Figure 2). 

**Figure 3:**
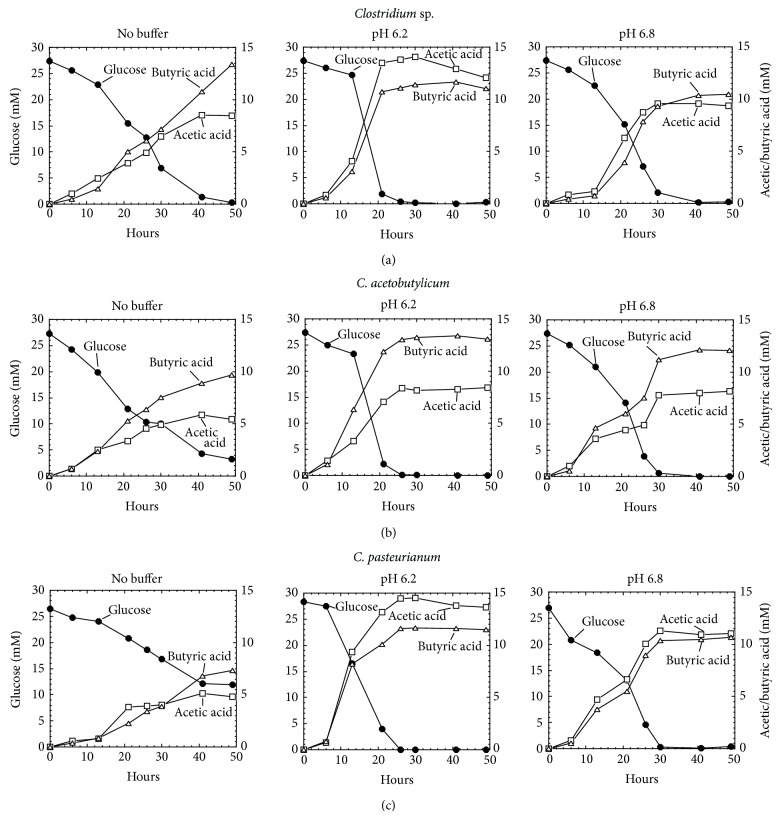
Glucose consumption and organic acid production by Clostridia grown in MSM medium supplemented with and without pH buffer.

Analysis of culture medium by HPLC showed that all these strains produced acetic acid and butyric acid (Figure 3). Glucose consumption and production of the organic acids were influenced by the initial pH of the medium; buffered medium at pH 6.2 showed the rapid glucose consumption and production of acetic and butyric acids. The maximum consumption of glucose was concurrently accompanied by reaching the production peak of butyric acid in all strains assayed. In the case of *Clostridium* sp., glucose consumption reached 100% completion at 50, 30, and 40 h in unbuffered, buffered medium at pH 6.2 and 6.8, respectively. Concurrently, butyric acid production attained its peak at the same time point as that of 100% glucose consumption, and the highest concentration measured was 13.5, 11.5, and 10.5 mM in unbuffered, buffered medium at pH 6.2 and 6.8, respectively. By contrast, acetic acid production reached its peak at 40, 30, and 30 h and the corresponding concentration measured was 8.5, 14, and 9.5 mM, in unbuffered, buffered medium at pH 6.2 and 6.8, respectively. Similarly, in the case of *C. acetobutylicum*, maximum consumption of glucose was reached at 50, 26, and 40 h in unbuffered, buffered medium at pH 6.2 and 6.8, respectively. At these time point, butyric acid production attained its peak and the concentration measured was 9.5, 13.5, and 12 mM in unbuffered, buffered medium at pH 6.2 and 6.8, respectively. Acetic acid production reached its peak at 42, 26, and 30 h and the corresponding concentration measured was 6, 8.5, and 8 mM in unbuffered, buffered medium at pH 6.2, and 6.8, respectively. In the case of *C. pasteurianum*, maximum consumption of glucose was approached at 50, 26, and 30 h in unbuffered, buffered medium at pH 6.2 and 6.8, respectively. At the same time point, butyric acid production attained its peak and the concentration measured was 7.5, 11.5, and 10.5 mM in unbuffered, buffered medium at pH 6.2 and 6.8, respectively. For this strain, acetic acid production reached its peak at 42, 26, and 30 h, and the corresponding concentration measured was 5, 14.5, and 11.5 mM in unbuffered, buffered medium at pH 6.2 and 6.8, respectively.

*C. sphenoides* showed no increase in cell density and growth after adding pH buffers to either MSN or SCM medium (data not shown). Further, HPLC measurements revealed that this strain only generated acetic acid when cultured in both SCM and MSM (data not shown).

We also observed that, when the medium was buffered at pH 7.2 and above (7.5), *Clostridium* sp. did not grow; the other three strains grew slowly at pH 7.2, but did not grow above 7.5 (data not shown).

### 3.2. Hydrogen (H_2_) Consumption Results in U(VI) and Fe(III) Reduction of Clostridia

**Figure 4:**
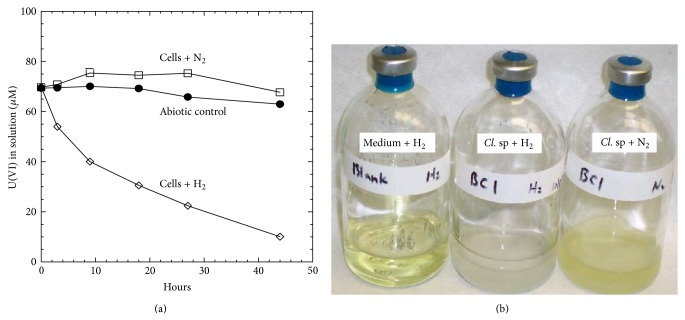
Hydrogen-driven reduction of U(VI) (a) and Fe(III) (b) by *Clostridium *sp. BC1.

We demonstrated that gaseous hydrogen, in the presence of clostridia cells, could result in both U(VI) and Fe(III) reduction (Figure 4). Cultured clostridia cells were spun down, washed, and then resuspended in carbon-free MSN medium in a serum bottle under anaerobic conditions. Thereafter, headspace gases were replaced with pure hydrogen or nitrogen gas. When hydrogen was provided as the headspace gas, either uranium(VI) or iron(III) reduction occurred in the presence of whole cells of *Clostridium *sp. BC1. This is in contrast to experiments which used nitrogen as the headspace gas. Without whole cells, hydrogen alone did not result in either uranium(VI) or iron(III) reduction, suggesting that hydrogenase indeed mediated both the uranium(VI) and iron(III) reduction using hydrogen as an electron donor (Figures 4(a) and 4(b)). Both the gas pressure and pH in the bottle containing bacterial cells with hydrogen decreased after overnight incubation. Using other Clostridia strains as testing material, similar results were also obtained (data not shown).

### 3.3. Copper(II) Inhibits Uranium Reduction

**Figure 5:**
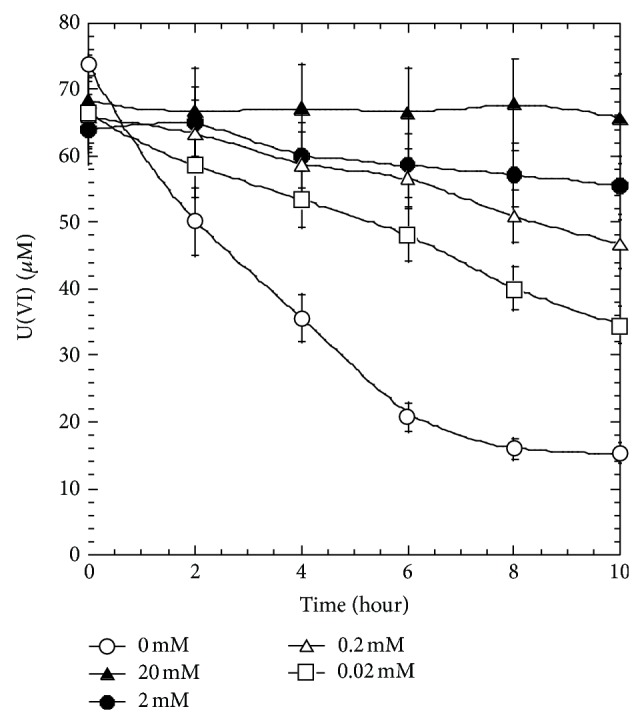
Effect of addition of Copper(II) on uranium reduction by *Clostridium *sp. BC1.

We found that Cu(II) strongly inhibit U(VI) reduction of Clostridia. When concentration of Cu(II) reached 20 mM, the U(VI) reduction activity by BC1 was 100% inhibited (Figure 5). The inhibition effect of Cu(II) is not relevant with the U(VI) form used (UO_2_(NO_3_)_2_ or U(VI)-citric acid complex) (data not shown). In terms of inhibition effect on uranium reduction, no difference was observed between simultaneous and stepwise addition of Cu(II) and U(VI) into assay (data not shown), suggesting that the inhibition effect of Cu(II) is immediate and most likely occurs at enzymatic level. 

### 3.4. Iron Deficiency Affects U(VI) Reduction by Clostridia

**Figure 6:**
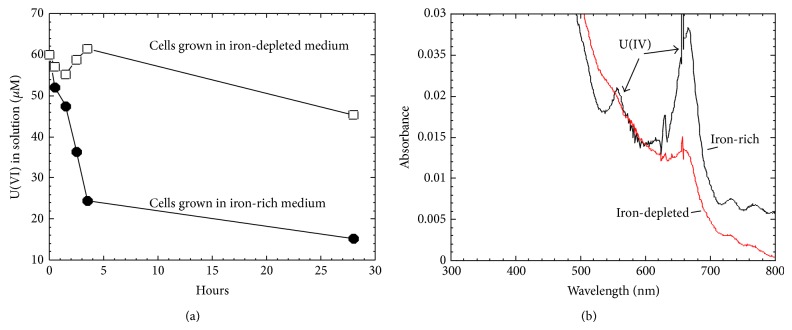
Iron deficiency affects U(VI) reduction by *Clostridium *sp. BC1. (a) Dynamic curve of U(VI) reduction; (b) UV-vis visualization of reduced U(IV).

*Clostridium. *Cells cultured in iron deficient media compromised its capability for uranium reduction (Figures 6(a) and 6(b)). Compared with iron-rich medium which contained 10 *μ*M Fe(II), BC1 cells cultured in iron-depleted medium containing <0.01 *μ*M, Fe(II) performed much poorer in U(VI) reduction to U(IV) during the time period of assay.

## 4. Discussion 

Previously, we demonstrated that *Clostridium* sp. BC1 and *C. sphenoides* can reduce uranium [15, 22]. Later, we expanded this list to *C. acetobutylicum* and *C. pasteurianum* that were identified many years ago and have been widely used in basic and applied studies [17]. For instance, ATCC 824, the type strain of species *C. acetobutylicum,* was isolated in 1924 from garden soil in Connecticut [23] and is one of the best-studied solventogenic clostridia used to develop an industrial starch-based acetone, butanol, and ethanol (ABE) fermentation process [8]. The entire genome of that strain was sequenced already [24]. Our study found that both *C. acetobutylicum* and *C. pasteurianum* can reduce uranium so bolstering our previous conclusion that this ability is a common phenomenon among clostridia bacteria [17]. We demonstrated that not only all of the clostridia tested are able to reduce U(VI) to U(IV), but also there are considerable differences in the extent of their ability to do so [17]. The extent for uranium reduction varies among clostridia strains and that pH of medium strongly influences the dynamic process of uranium reduction. Without a buffer supplement, our experiments showed that the pH of the cultures could drop to 3 to 4 when the late-log phase of growth was reached; then, only the *Clostridium* sp. culture reduced U(VI) to U(IV) well, while the other three strains could not do so, or performed poorly [17]. In this study, we demonstrated that *Clostridium* sp. was superior in terms of growth and gas production. Thus, it seems reasonable to attribute the poorer performance of strains other than *Clostridium* sp. BC1 in U(VI) reduction to suboptimal growth. Meanwhile, our findings also exemplified that *Clostridium* sp. has higher tolerance to harsh environmental conditions, such as low pH, as well as a stronger ability for fermentation under such conditions, especially in reducing U(VI) in acidic environments. Since this strain was isolated from an acidic metal-contaminated site [25], its better adaptation to acidic environments is unsurprising. 

However, adding the pH buffer to cultures of *C. sphenoides* caused no improvement in its growth. Nevertheless, the reduction rate of U(VI) by this strain was much better at a near-neutral pH, and its highest rate mostly closely approached that of *Clostridium* sp. [17]. Thus, an optimal pH alone apparently is important to U(VI) reduction by clostridia, even though it does not necessarily improve fermentation efficiency at the same time. We found that a suboptimal pH could compromise the ability of clostridia to reduce U(VI): we suggest that the underlying mechanism for this phenomenon is as follows. Since most physiological reactions ideally occur at near-neutral pH, organisms developed a variety of mechanisms to maintain a near-neutral cytoplasmic pH. Even those acidophilic bacteria that grow best at ~pH 3 maintain a near-neutral cytoplasmic pH and possess a membrane potential (ΔΨ) with an orientation reversed from that found in neutrophilic bacteria [26]. For gram-positive bacteria, including Clostridia, proton efflux through proton pumps, such as F_1_F_0_ATPase, is the major means of raising internal pH [27]. Goodwin and Zeikus [28] demonstrated that physiological adaptations of anaerobic bacteria to low pH often are a competitive process for hydrogen production. Thus, we speculate that the physiological adaptations of clostridia to suboptimal pH also compete with U(VI) reduction. Under suboptimal conditions, the bacterial response to the stressful environments becomes their priority, and more energy than normal is consumed in activities, such as amino acid metabolism, transcriptional regulation and signal transduction, transport, maintaining cell-membrane structure, and protection against oxidative stress, as was suggested by a transcriptome analysis of *Shewanella oneidensis* cells exposed to acidic and alkaline pHs [29]. Meanwhile, under the same conditions, other activities become secondary ones, such as U(VI) reduction, and little energy is allocated to them.

The mechanisms of uranium(VI) reduction by anaerobic respiratory bacteria, including *Desulfovibrio*, *Geobacter*, and *Shewanella*, are being extensively investigated [5, 6]. However, although uranium reduction by Clostridia is established, the mechanisms underlying the reaction remain unclear. Our previous results showed that it was an enzymatic process since it happened only in the presence of growing or resting cells; neither the organic-acid metabolites generated nor the extracellular components of the culture, nor heat-killed cells could reduce uranium anaerobically [14]. Previous conjectures were that the reducing power generated from fermentation, such as that of glucose, caused uranium reduction. Thus, Petrie at al. [18] demonstrated that glucose amendments of the growth medium enriched the numbers of gram-positive spore-forming bacteria, and since some of the highest rates of U(VI) reduction *in situ* occurred upon amendment, that fermentative processes were involved [30]. Our previous study showed that the optimizing conditions for fermentation resulted in better U(VI) reduction support this hypothesis. Indeed, improving fermentation conditions by supplementing the medium with a pH buffer increased the U(VI) reduction rate of Clostridia strains [17]. Apparently, the efficiency of fermentation is positively related to the U(VI) reduction rate of individual strains. 

 In this study, we demonstrate that the hydrogen metabolism could play an important role in both uranium(VI) and iron(III) reduction by *Clostridium* sp. When hydrogen gas (H_2_) was provided in the headspace of the serum bottle, either uranium(VI) or iron(III) reduction occurred in the presence of whole cells without carbon source. This is in contrast to the introduction of nitrogen gas (N_2_) into the headspace. In the absence of whole cells, hydrogen alone could result neither uranium(VI) nor iron(III) reduction, suggesting that a hydrogenase mediated both the uranium(VI) and iron(III) reduction using hydrogen as the electron donor (Figure 4). Evidence supporting this hypothesis also came from Cu(II) inhibition effects on uranium reduction of clostridia (Figure 5). Since Cu(II) is a documented hydrogenase inhibitor [31], the inhibition of U(VI) reduction is most likely through the inhibition of hydrogenase activity of Clostridia cells. Hydrogenase mediated metal reduction also occurred in anaerobic metal respiring bacteria [32]. Our experiment also showed that *Clostridium* cells cultured in iron deficient media compromised its capability for uranium reduction (Figure 6). It is also noteworthy that the deficiency in ferrous ions also affected hydrogen production (data not shown). Still, our recent results showing that methyl viologen (MV) addition affects hydrogenase activity with a significant reduction in hydrogen production also agree with this hypothesis [33]. Taken together, one scenario explaining molecular mechanisms of uranium(VI) reduction by clostridia is emerging and is described as below.

**Figure 7:**
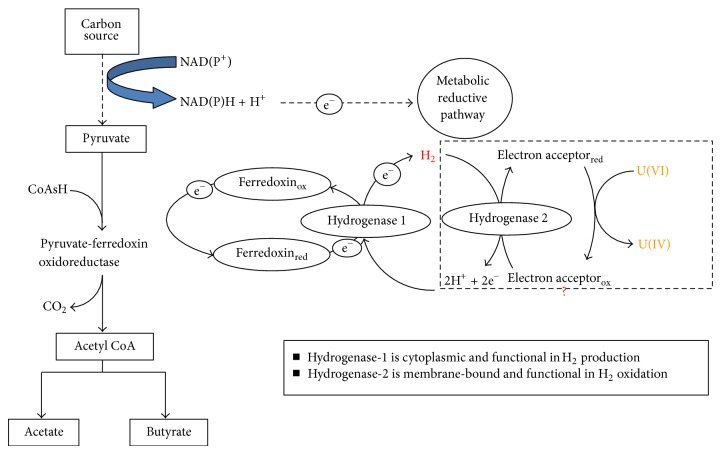
A hypothetic scheme illustrating clostridia fermentation pathway leading to hydrogen production and its roles in uranium reduction.

Clostridia dispose of excess electrons generated during fermentation by producing hydrogen. During the fermentation process, bacteria that grow at the expense of diverse carbon sources often depend on ferredoxin or flavodoxin for essential oxidation-reduction reactions [34]. The metabolic pathway of hydrogen production in clostridia is summarized in Figure 7.

As shown in Figure 7, Clostridia use ferredoxin (Fd) to oxidize sugars and other organic matter through pyruvate to produce carbon dioxide, acetate, and butyrate. Ferredoxins are acidic, low molecular weight, soluble iron-sulfur proteins found in various organisms and act as multifunctional electron carriers in diverse redox systems [34]. Flavodoxin can replace ferredoxin in most of the reactions. Flavodoxin is a flavin mononucleotide-binding redox protein with an open twisted alpha/beta structure consisting of five parallel beta-sheets connected by alpha-helices which surround the sheets in the 3D structure. Ferredoxins or flavodoxins are essential components of the electron transport chains of clostridia. However, they do not themselves possess enzymatic activity in these reactions. Instead, they interact with specific dehydrogenases and reductases that handle the substrates to be oxidized or reduced. Hydrogenase, a molecular hydrogen evolving enzyme, receives electrons from pyruvate oxidation through up to seven Fd clusters to produce hydrogen (H_2_) by the reaction as follows: 2H^+^ + 2e^−^ *↔* H_2_ [1]. In some strains of clostridia, the presence of nitrogenase complicated hydrogen metabolism. In the presence of nitrogen and absence of ammonia, nitrogenases catalyze the production of hydrogen and ammonia at the expense of ATP. Without nitrogen, nitrogenases can function as 100% hydrogenase to produce hydrogen in a process that requires ATP and electrons from biomass.

 The molecular basis for biological hydrogen production is dependent upon the presence of hydrogen-producing enzymes. At present, there are three known enzymes carrying out such reactions: Fe-hydrogenase, Ni-Fe hydrogenase, and nitrogenase. Iron-hydrogenase was first isolated from *Clostridium pasteurianum *[10]. It is also found in other strict anaerobic bacteria such as *Desulfovibrio vulgaris* [35, 36], as well as in some green algae such as *Chlamydomonas reinhardtii* [37], and several eukaryotic protists such as *Trichomonas vaginalis* [38]. A Fe-hydrogenase may exist as a distinct monomer or heteromer [39]. Generally, the cytoplasmic Fe-hydrogenase (Hydrogenase-1) functions as the generator of hydrogen by removing excess reducing equivalents during fermentations of strict anaerobic bacteria, whereas the periplasmic Fe-hydrogenase (Hydrogenase-2) functions in hydrogen oxidation [40]. Fe-hydrogenase contains a unique complex Fe-S centers in which one of the Fe atoms is complexed with CO and CN [41]. Compared with nitrogenase and Ni-Fe hydrogenase, Fe-hydrogenase is highly efficient in hydrogen production.

As shown in Figure 7, we postulate that both Hydrogenases 1 and 2 are involved in uranium reduction of clostridia. While Hydrogenase 1 functions as the generator of hydrogen by removing excess reducing equivalents during fermentations, Hydrogenase 2 functions in hydrogen oxidation and more directly involved in uranium reduction. It is not clear how the hydrogenases of clostridia consume hydrogen and transfer electrons to iron(III) or uranium(VI). In addition to hydrogenase, it is not known if additional proteins are involved in this process. One of such other possible candidates conducting direct electron transfer is cytochrome protein. Cytochromes are generally membrane-bound proteins that contain heme groups and carry out electron transport. Many cytochromes (c-type, b-type) of dissimilatory metal reducing bacteria (DMRB) were shown to be relevant to metal reduction [42, 43]. The presence of cytochrome in clostridia has been documented previously [44]. From the annotated genome sequence of *Clostridium acetobutylicum* (ATCC 824) [24], around 30 cytochrome-like proteins have been identified. We also postulate that iron depletion could result in the shutdown or downregulation of hydrogenase synthesis as well as other iron-containing proteins like ferredoxin. Meanwhile, as a substitution, the expression of some noniron proteins such as flavodoxin could be upregulated. Due to this shift in protein synthesis, clostridia compromise their metabolism of hydrogen production, as well as their capability for uranium reduction. Note that our proposed model herein does not necessarily exclude possible other pathways and other components also used by Clostridia for uranium reduction, such as spore-mediated metal reduction [45]. 

## 5. Final Remarks

With their widespread occurrence in soils, sediments, and low-level radioactive wastes, Clostridia could play a significant role in the *in situ* reduction of uranium and other metals particularly at acidic pH and in nitrate-rich environments, as suggested by a number of studies [18, 20, 21, 25, 30]. However, compared with anaerobic respiratory bacteria including *Desulfovibrio*, *Geobacter*, and *Shewanella*, molecular mechanisms underpinning uranium(VI) reduction to uranium(IV) are still not very clear. Future study toward this direction should be encouraged and we expect more detailed mechanisms of uranium reduction by Clostridia will be revealed in the future.
